# Ensemble classification of integrated CT scan datasets in detecting COVID-19 using feature fusion from contourlet transform and CNN

**DOI:** 10.1038/s41598-023-47183-9

**Published:** 2023-11-16

**Authors:** Md. Nur-A-Alam, Mostofa Kamal Nasir, Mominul Ahsan, Md Abdul Based, Julfikar Haider, Marcin Kowalski

**Affiliations:** 1 Department of Computer Science & Engineering, Mawlana Bhashani Science and Technology University, Tangail, 1902 Bangladesh; 2 Department of Computer Science, University of York, Deramore Lane, York, YO10 5GH UK; 3 Department of Computer Science & Engineering, Dhaka International University, Dhaka, 1205 Bangladesh; 4 Department of Engineering, Manchester Metropolitan University, Chester St, Manchester, M1 5GD UK; 5 Institute of Optoelectronics, Military University of Technology, Gen. S. Kaliskiego 2, Warsaw, Poland

**Keywords:** Signs and symptoms, Respiratory signs and symptoms, Computational biology and bioinformatics, Classification and taxonomy, Computational models, Data processing, Machine learning

## Abstract

The COVID-19 disease caused by coronavirus is constantly changing due to the emergence of different variants and thousands of people are dying every day worldwide. Early detection of this new form of pulmonary disease can reduce the mortality rate. In this paper, an automated method based on machine learning (ML) and deep learning (DL) has been developed to detect COVID-19 using computed tomography (CT) scan images extracted from three publicly available datasets (A total of 11,407 images; 7397 COVID-19 images and 4010 normal images). An unsupervised clustering approach that is a modified region-based clustering technique for segmenting COVID-19 CT scan image has been proposed. Furthermore, contourlet transform and convolution neural network (CNN) have been employed to extract features individually from the segmented CT scan images and to fuse them in one feature vector. Binary differential evolution (BDE) approach has been employed as a feature optimization technique to obtain comprehensible features from the fused feature vector. Finally, a ML/DL-based ensemble classifier considering bagging technique has been employed to detect COVID-19 from the CT images. A fivefold and generalization cross-validation techniques have been used for the validation purpose. Classification experiments have also been conducted with several pre-trained models (AlexNet, ResNet50, GoogleNet, VGG16, VGG19) and found that the ensemble classifier technique with fused feature has provided state-of-the-art performance with an accuracy of 99.98%.

## Introduction

COVID-19, caused by the severe acute respiratory syndrome coronavirus 2 (SARS-CoV-2), quickly spread throughout China in December 2019 and the rest of the world. By July 2022, there had been over 576 million confirmed cases worldwide, with 6.41 million deaths^1^. COVID-19 has been declared a global pandemic by the World Health Organization (WHO) and when the disease progressed to the severe/critical stage, approximately 60% of the patients died^2^. Massive alveolar injury and gradual respiratory failure are believed to be the leading causes of death. Coronavirus is a virus that causes a taint in sinuses, nose, or upper throat leading to pneumonia, respiratory failure, liver problem, heart problem, septic shock, and eventually death. Like several RNA viruses, SARS-CoV-2 is one of the most dangerous diseases with several variants and no obvious symptoms. As a result, rapid and precise COVID-19 screening and diagnosis is critical for planning early therapies, stopping the transmission path, and developing clinical schemes to enhance prognosis^3^.

COVID-19 can be diagnosed in two ways. The first one is a real-time polymerase chain reaction (RT-PCR) test for nucleic detection. Clinical diagnosis, discharge assessment, and recovery follow-up have all benefited from the use of RT-PCR. However, the sensitivity of RT-PCR from swab samples is limited, which could lead to a lot of false negatives^4^. Chest medical imaging either by X-ray or computed tomography (CT) is the second method for detecting COVID-19. COVID-19 has been linked to many tiny patches and ground glass shadows on CT in clinical investigations. In terms of pathology, CT image can provide precise information that can be used to provide a quantitative assessment of the pulmonary abnormalities that could have prognostic consequences^5^. In general, despite its great sensitivity (97%), CT is not suited for large-scale screening because of its relatively high cost^6^. Furthermore, CT emits a significant dosage of radiation, which is hazardous to the human body. CT can be utilized to provide reliable clinical diagnoses; however, it is not recommended for clinical applications that need recurrent data collection. Another medical imaging technique for detecting COVID-19 is X-ray. As an X-ray cannot provide 3D information like a CT scan, radiologists typically utilize it as a screening tool before a CT diagnosis. A large number of studies so far have focused on the CT diagnosis^5–7^, while the X-ray diagnosis has received relatively less attention^8^.

For the purpose of differentiating between COVID-19 and other CT scans, Polsinelli et al.^9^ suggested a CNN design (SqueezeNet CNN based) that achieved an accuracy of 85.03%. They assessed their model utilizing data from Italian dataset (100 COVID-19 CT scans) and Zhao et al. dataset (360 COVID-19 CT scans, 397 healthy/others). The key advantage of their model was that it consumed less average categorization time on both high-end computers (7.81 s per CT image) and medium-end computers (1.25 s per CT scan). However, by utilizing effective pre-processing approaches, the performance of their preferred scheme can be significantly enhanced.

Basu et al.^10^ suggested a two-stage approach (feature extraction followed by feature selection) in their work to detect COVID-19 from the CT scan images. CNN models (DenseNet, ResNet, and Xception) were employed in the feature extraction phase to produce a feature vector from the input images. To remove unimportant characteristics from the acquired feature vectors, a combination of the global optimization algorithm HS and the local optimization algorithm AβHC was used. For the training and testing of their proposed methodology, they employed two separate datasets from the SARS-COV-2 CT-Scan Dataset5 with 2482 and 2926 CT images. On the two datasets, the proposed technique yielded the best accuracy ratings of 97.30% and 98.87%, respectively. Their main drawback of the approach was that it was not capable of diagnosing COVID-19 positive from the CT scans at the very early phases of the disease. This could be mostly due to lack of substantial artifacts in the images. As a result, the CNNs employed in their work were unable to locate the characteristic features.

Kandati and Gadekallu^11^ proposed system to accurately detect chest lesions resulted from COVID-19 infection by combining two CNN models: Federated Learning (FL) and particle swarm optimization algorithm (PSO). The Federated Particle Swarm Optimization approach was tested on a multidimensional COVID-19 infected chest lesion image dataset and the chest X-ray (pneumonia) dataset from Kaggle’s repository. The proposed model achieved 96.15% prediction accuracy in detecting COVID-19 infected chest lesions.

Karthik et al.^12^ designed a regression-based method for COVID-19 severity rating using a deep learning network in order to diagnose the severity of a patient’s medical condition from the CT scan. A variety of cues are encoded into hierarchical attention layers which used a customized CNN that operated as a multi-stage analysis tool. To provide a solid encoded depiction for the decoder, multi-scale features were precisely extracted and merged. After applying cross-channel correlation and compressing the structural and semantic information in the fused contextual map into a global reference encoding, the transformed feature set was compared to the baseline CT scan through a non-local attention mechanism that transcribed the lesion locations. The suggested design has a 0.84 R-squared score, according to the experimental analysis on the MosMed dataset (1110, 3D CT scan images). One standout achievement of this approach was the design of explicit guidance to modulate the attention head.

Aversano et al.^13^ developed a novel ensemble-based method that took advantage of transfer learning utilizing pre-trained deep networks morphed with a genetic algorithm, associated with an ensemble architecture for the categorization of clustered images of lung lobes. Their research was supported by a new dataset that was created by combining several earlier datasets. Considering that the F1-score barely ranged from 0.94 to 0.95, the effectiveness of the ensemble trained on the integrated dataset was fairly steady. The primary weakness of this study was the unbalanced dataset with 780 COVID-19 images and 14,520 nonCOVID-19 images.

A new automatic method for COVID-19 screening with the chest CT scans was introduced by Zhao et al.^14^. To initially extract the pulmonary parenchyma, the SP-V-Net image deformation-based segmentation model, which included a 3D V-Net for CT image segmentation and a STN for output restriction and refinement, was developed. The features extracted from the segmented lung lobes were employed for quantitative analysis with a high applicability to identify COVID-19 infection. Their study included self-collected 112 CT scans in total. An AUC of 0.9470 was attained by their proposed COVID-19 classification model using statistically representative radiomic features.

The contrastive multi-task convolutional neural network (CMT-CNN) proposed by Li et al.^3^, transformed each image through a sequence of augmentations. The model was then tuned to incorporate representations of the identical images that were similar while the distinct images that were dissimilar in a latent space. In this manner, the spread-out features of the data were maintained and the CMT-CNN was capable of making recommendations that were invariant to transformation. They performed experiments employing two datasets: a CT dataset (4758 samples) and an X-ray dataset (5821 samples), which were put together using both open and self-collected archives. Results from the research indicated that their methodology significantly improved accuracy for DL models on CT (by 5.49–6.45%) and X-ray (by 0.96–2.42%) images without the need for any additional annotation.

Amyar et al.^15^ introduced a new multitask deep learning model where the architecture was composed up of a common encoder for disentangled feature representation with three tasks, two decoders, and a multi-layer perceptron for reconstruction, segmentation, and classification, respectively. A self-created dataset of 1369 patients was used to assess the suggested model and compare it to other alternative image segmentation methods. According to the results, the segmentation had a dice coefficient greater than 0.88 and the classification had an area under the ROC curve greater than 97%.

For automated COVID-19 lung segmentation and severity assessment in 3D chest CT scans, He et al.^16^ proposed a synergistic learning framework. They created a multi-task multi-instance deep network (M2UNet) to assess the severity of COVID-19 patients and segment the lung lobe at the same time, where the context data supplied by the segmentation could be utilized to improve the performance of the severity evaluation. To begin with, they depicted each input image by a bag to deal with the challenging problem that the severity was attributed to the local infected regions in the CT scan image. In M2UNet, a hierarchical multi-instance learning technique was also suggested for severity evaluation. Through experimental analysis of their prepared dataset (666 CT scans), they demonstrated that their method outperformed several cutting-edge techniques by obtaining an accuracy of 98.5%.

In order to identify COVID-19 utilizing relatively small-sized CT images, Li et al.^17^ presented a deep learning methodology based on transfer learning. Their suggested approach made use of the transfer learning principles, which moved information from one or more source tasks to a target domain when the latter had less training sets. CheXNet was employed for COVID-19 identification by fine-tuning the network weights on the limited dataset for the objective goal. Evaluation was carried out on the freely accessible COVID-19-CT dataset (349 CT scans of 216 COVID-19 patients). According to the experimental findings, their method provided good performance in comparison to six state-of-the-art approaches by achieving an accuracy of 87%. However, their network design and optimizer still have scope for further development. In addition to the challenges, they continued to encounter data dependence, one of the most serious issues with deep learning makes it impossible to train the models in some specialized fields, particularly at the early stages of the COVID-19 spread when attempting to capture the characteristics of COVID-19 and Non-COVID-19. Table 1 summarizes existing image-based system methodologies in COVID-19 detection and their limitations.

**Table 1:** Existing methods for COVID-19 diagnosis.

References	Methodology	Finding	Limitation
Polsinelli et al.^9^	A light convolutional neural network (CNN) design (SqueezeNet)	Classify COVID-19 and normal images, used 460 CT scan images and accuracy 85.03%	Low classification accuracy and high computational complexity, works for high-resolution images, using small datasets (460)
Basu et al.^10^	CNN-based feature extractor + meta-heuristic optimization algorithm, harmony search (HS), combined with a local search method, adaptive β-hill climbing (AβHC) for feature selection	Classify COVID-19 positive and negative, containing 2926 CT scan datasets, accuracy 98.87%	It may not be able to detect COVID-19-positive from CT scans at the very early stage of infection
Kandati and Gadekallu^11^	Combined two CNN models federated learning (FL) and particle swarm optimization algorithm (PSO)	Early detection of chest lesion, used 317 CT scan images, accuracy 96.17%	Cannot detect low-resolution images of COVID-19, limited datasets
Karthik et al.^12^	Data preprocessing + Data Augmentation + multi-scale features + regression learning	CT-based severity assessment for COVID-19. Used 1110 3D CT scan images, accuracy 84.30%,	False detection for the noise and artifact-affected images
Aversano et al.^13^	Dataset merging and clustering + ensemble classifier (VGG, Xception and ResNet)	To detect COVID-19 or normal CT scan images, used 23,398 CT scan images, accuracy 95.10%	Reduced number of images used to build the joint dataset
Zhao et al.^14^	Data preprocessing + segmentation (V-Net) + SVM classifier	Diagnosis of COVID-19 infection on chest CT images, used 212 CT scan images, accuracy 94.70%	Limited data (212)
Li et al.^3^	Transformed image + feature extraction + contrastive learning + contrastive multi-task convolutional neural network for classification	Classify COVID-19 positive and negative images, used 4748 CT scan images, accuracy 93.90%	Demands substantial memory space, restricts the batch size, medical imaging data is sparse and expensive to label
Amyar et al.^15^	Segmentation + U-Net classification	Classification and segmentation of COVID-19 image, used 1369 CT scan images, accuracy 94.67%	Noisy image
He et al.^16^	2D image patches + feature embedding + classifier (M^2^UNet)	Detect positivity and severity of COVID-19, used 666 Ct scan images, accuracy 98.50%	High computational time. classification more complex
Li et al. 202^17^	Preprocessing + feature extractor + modified CheXNet	Classify COVID-19 or normal image, used 1212 X-ray images, accuracy 87%	Small-sized training datasets

According to the findings of the above studies, four key challenges in COVID-19 detection research have been identified: (a) segmentation of COVID-19 image region, (b) extraction of discriminating characteristics, (c) detection or classification approach based on the retrieved features and (d) limited number images in the dataset. Many region clustering algorithms for segmentation have been offered by the researchers, however, the best one is still yet to be found. On the other hand, feature extraction methods can be based on a single strategy or a combination/fusion of strategies. In most cases, the fusion approach yields better results. Besides, selecting an appropriate detection method can be challenging as the number of choices is too many. Hence, an improved method capable of executing region-based segmentation, fusing features extracted by more than one technique, selecting appropriate features, and conducting accurate classification from a large number of images would be required to overcome the existing limitations.

As artificial intelligence (AI) has proven to have outstanding capability in the autonomous diagnosis of COVID-19 based on CT, thanks to deep learning’s strong representational learning ability. AI offers several benefits: (1) Make a speedy diagnosis, especially if the medical system is overburdened. (2) Lighten the load on radiologists and (3) Assist underdeveloped areas in getting a proper diagnosis. Most critically, as a new pandemic, there is a lack of systematic consensus on the sensitivity and particular signs of COVID-19. AI can develop discriminative features automatically based on the available data, which can help in identifying COVID-19 from other pneumonia^18^. Despite the success of AI in COVID-19 CT diagnosis, the models’ generalization is still lacking and must be enhanced further to improve the detection accuracy.

This paper aims to develop a machine learning (ML) based automatic system that detects COVID19 either positive or negative from the CT scan images and provides better output compared to the existing methods. The main contributions have been provided as follows.

1. The proposed method has developed a new database by collecting two different categories of CT scan images consisting of normal and COVID-19 from three publicly available major data sources^19–21^.
2. A modified region-based clustering method has been applied to segment the whole CT scan image leading to a better classification result.
3. A fused feature vector has been proposed from two different feature extraction methods including contourlet transform and CNN.
4. Hybrid binary differential evolution (BDE) has been selected for obtaining Meta heuristic features from the fused feature vector and achieving optimized features.
5. A voting-based technique has been suggested for detecting COVID-19 using an ensemble of three base classifiers.

The rest of the paper is organized as follows: “Model development” section describes the methodology to detect COVID-19 using the CT image and deep neural network. “Results and analysis of the proposed method” section illustrates the experiments conducted with corresponding classification performance and model validation. “Discussion” section presents discussions on performance comparison with the existing methods, complexity analysis and limitations. Conclusions and future research directions are outlined in “Conclusion” section.

## Model development

### Proposed methodology

**Figure 1:**
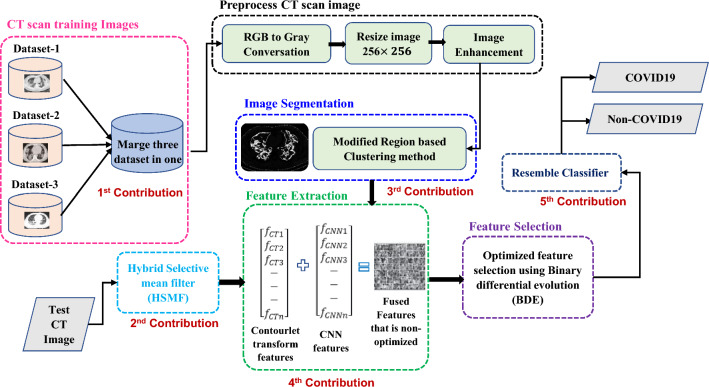
Proposed methodology for detecting COVID-19.

The architecture of the proposed model as shown in Fig. 1 considered CT scan images as the input to detect COVID-19 or non-COVID-19 images. The CT scan image datasets were collected and merged from three publicly available datasets. Since the dataset images were not of the same size, they were resized and merged. The images were then converted to grayscale from RGB. A modified region-based clustering method was proposed to segment the CT scan grayscale images. Furthermore, the model deliberated two feature extraction techniques including contourlet transform and CNN. Firstly, the contourlet transform method and secondly, the CNN feature extraction technique extracted feature vectors. These two vectors were fused in one feature vector, which was used as the input to train the classification model. The fused feature vector considered a large number of features that helped to accurately identify the COVID-19 or normal images. The system also proposed an authentic feature selection technique that extracted meta-heuristic features by using BDE. This optimized vector was subsequently used to recognize COVID-19 CT scan test pictures using an ensemble classifier.

The most important step in designing a computer-aided diagnostic (CAD) system for detecting COVID-19 at an early stage is the CT scan image segmentation^22^. In order to diagnose unusual disorders, segmentation is widely used in the area of medical images. Manual segmentation of the same medical images is possible. Image segmentation utilizing segmentation algorithms has a higher accuracy compared to manual segmentation. The original fuzzy c-means (FCM) algorithm^23^ works well for segmenting noise-free images, however, it fails to accurately segment the images with noise, outliers, or other imaging artifacts. The modified region-based clustering technique was used in this work to segment the CT images. The objective of the modified region-based clustering algorithm was updated to reduce the intensity of homogeneities by including spatial neighborhood information and altering the membership weighting of each cluster. The proposed segmentation algorithm has the following advantages: (a) propagates more homogeneous regions than other old fuzzy c-means algorithms, (b) manages noisy spots and (c) it is comparatively less sensitive to noise. These techniques have produced excellent output images with the simplest approach to isolate the objects from the background.

### Dataset used

A chest CT scan is a useful medical imaging tool for accurately diagnosing COVID-19 cases^24^. As the open repository had a limited quantity of CT scan images, thus the images from all three databases were integrated to form a new database for this work. A total of 11,407 CT images with 7397 images from COVID-19 class and 4010 images from non-COVID19 class. The training and testing phases included images of COVID-19 and non-COVID-19.

- The SARS-CoV-2 CT-scan dataset^19^ has 2482 CT scan images from 120 patients, including 1252 CT scans of 60 patients infected with SARS-CoV-2 from men (32) and females (28), and 1230 CT scan images of 60 patients who were not infected with SARS-CoV-2 but had other pulmonary disorders. The data of CT scan images was gathered from hospitals in Sao Paulo, Brazil. The CT scan images in this dataset are digital scans of printed CT tests, and there is no criterion for image size. The smallest CT scan images in the dataset are 324 × 412 pixels, while the largest CT scans are 484 × 456 pixels. In this dataset, the number of training and testing images are 1842 and 640 respectively.
- The original CT scans image of 377 people are included in this COVID-19 CT image dataset^20^. There are 1558 and 4826 CT scan images, respectively, belonging to 95 affected COVID-19 people and 282 normal people. The Negin Medical Center in Sari, Iran, provided this dataset. All the CT image sizes are 256 × 256 × 3. In this dataset, the number of training and testing images are 5594 and 790 respectively.
- These publicly available datasets are collected from authentic website^21^. This dataset contains a total of 2541 CT scan images with 1200 COVID-19 and 1341 non-COVID-19. In this dataset, a total of 1726 and 815 images are considered for the training and validation.

**Figure 2:**
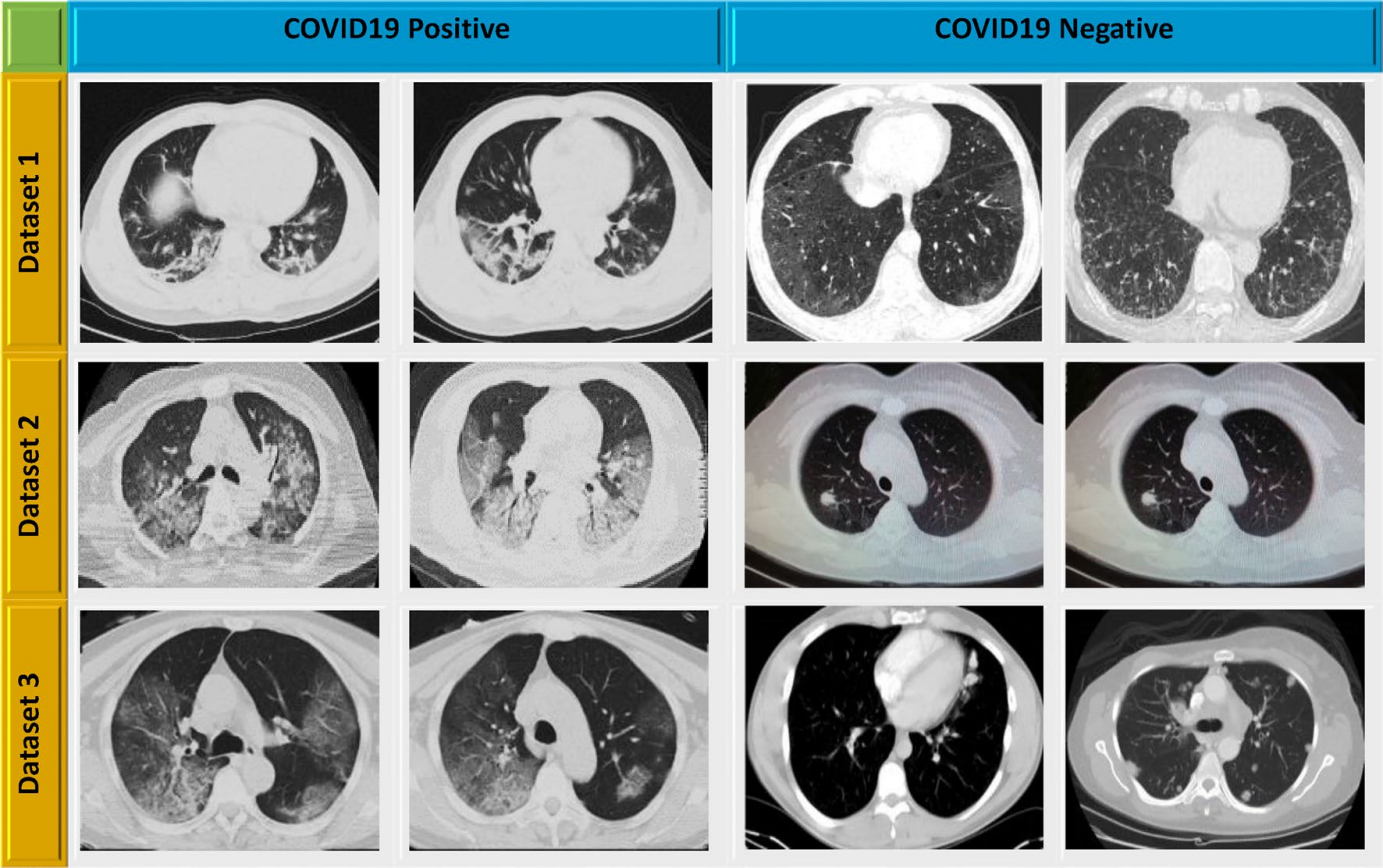
Sample CT scan images from three datasets.

As the open repository had a limited quantity of CT scan images, the images from all three databases were integrated to form a new database for this work. A total of 11,407 CT images with 7397 images from the COVID-19 class and 4010 images from the non-COVID-19 class. Figure 2 demonstrates sample CT scan images from each dataset. The training and testing phases included images of COVID-19 and non-COVID-19.

### Preprocessing

**Figure 3:**
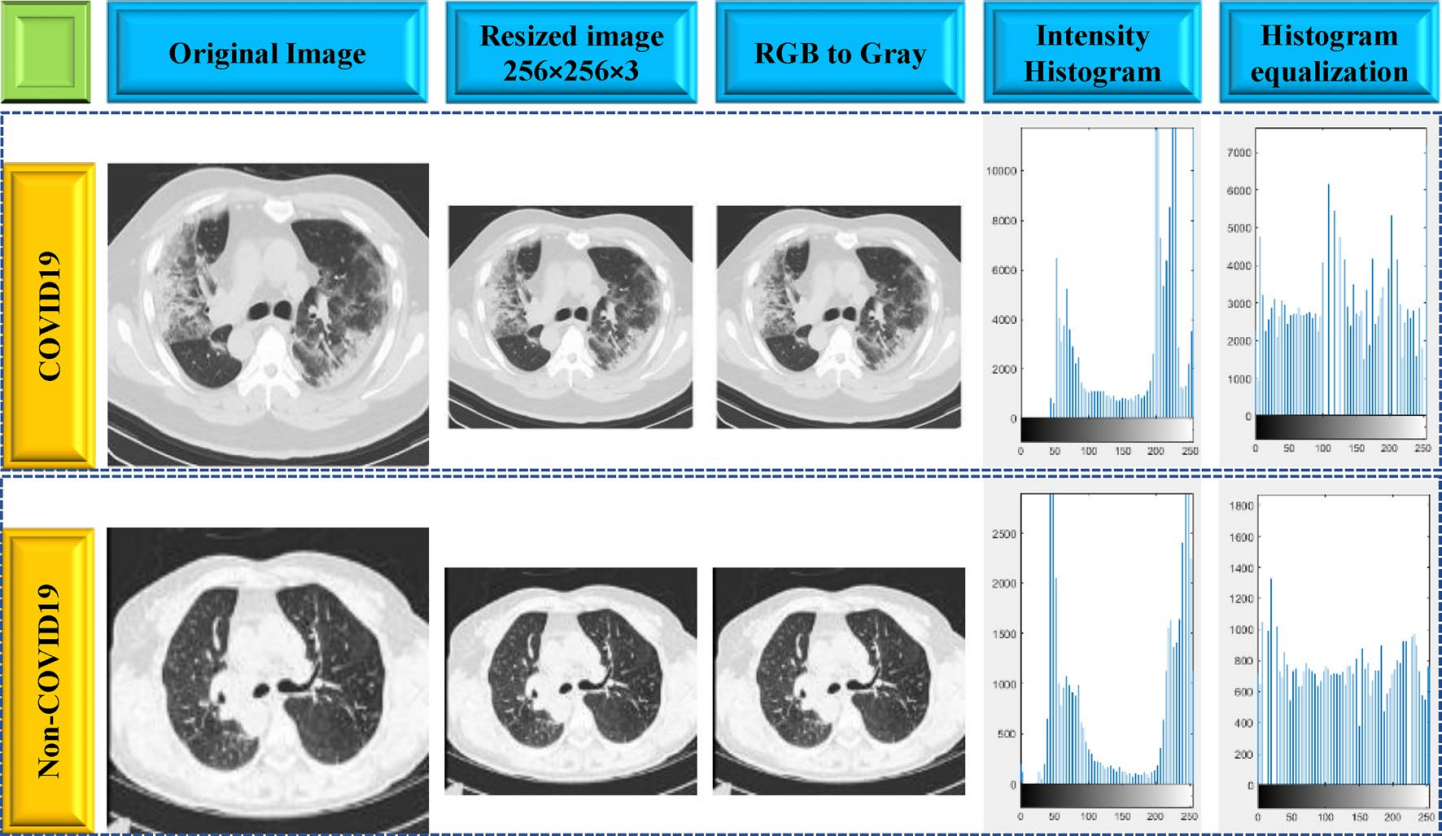
Preprocessing steps applied to the COVID-19 and non-COVID-19 images.

Image pre-processing is a key step in medical image processing to obtain meaningful information and appropriate classification by eliminating noisy or distorted pixels from each CT scan image. In this stage, the images were first resized to 256 × 256 pixels and transformed from RGB to grayscale images using the MATLAB function as the input for the model development. Color has no significance in detecting COVID-19 from the CT scan images hence grayscale images were employed during building the models to avoid any false classification and complexity. Grayscale images are simpler and easier to process than color images because they contain only one-color channel, which represents the intensity of the color for each pixel. Figure 3 displays the preprocessing steps employed in this work.

Histogram equalization, an image processing technique that is frequently used on CT scan images to improve image quality in black and white color scales. The input images and its contrast-enhanced (after histogram equalization) images are shown in Fig. 3 with the related histograms. Histogram equalization was achieved by efficiently spreading out the most frequent intensity values, extending the image intensity range. The adoption of a spatially variable histogram equalization technique seems to improve the visibility of anatomic structures in various clinical scenarios^25^. However, the technique increased the amount of noise and artifacts in the presented image.

### Modified region-based clustering techniques

The region-based clustering was employed to simplify the COVID-19 image region, which ensured less computational complexity and relatively accurate analysis. K-means, C-means, thresholding, morphology-based, edge-based, watershed, region-growing, and cluster-based approaches are among the various segmentation algorithms^26^. The authors of this paper proposed a cluster-based algorithm that segmented the image effectively and provided a better performance in terms of measuring evaluation matrices SSIM (structural similarity index), PSNR (peak signal to noise ratio) and RMSE (root mean square error) scores.

**Figure 4:**
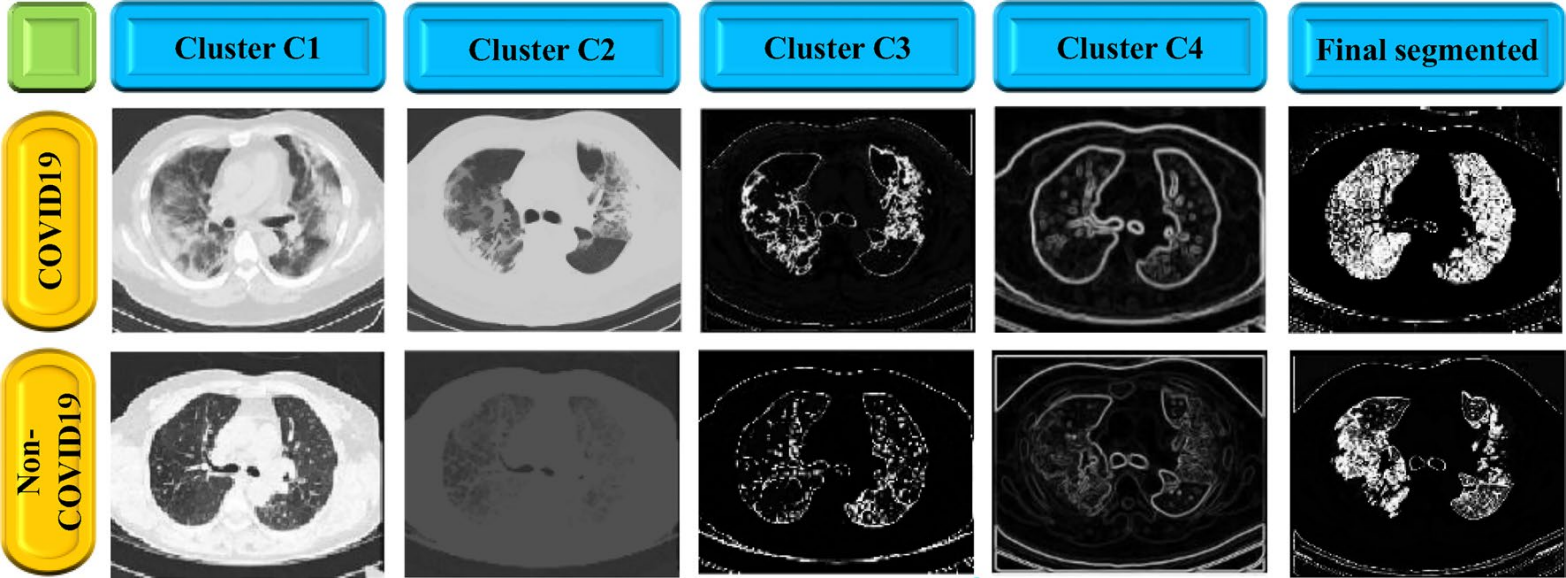
Applied modified region based clustering method for COVID19 and non-COVID19 image segmentation.

The proposed segmentation method partitioned the COVID-19 image into four clusters (C1 to C4) as gray matter (GM), cerebra-spinal fluid (CSF), white matter (WM), the necrotic focus of glioblastoma multiforme (GBM). The proposed segmentation technique employs an iterative process to locate the cluster region. In each iteration, the cluster’s centroid is modified to reduce the distance between pixels and the centroid. The mean brightness of all pixels within a cluster and the distance are obtained by using Eqs. (1) and (2) respectively. The COVID-19 segmentation process is depicted in Algorithm 1.

1 $${\mu }_{k}= {C}_{k} \sum_{i=0}^{N}\frac{{Z}_{i}}{N},$$

2 $$r= \left|{\mu }_{k}-{x}_{i}\right|,$$

where ${\mu }_{k}$ is the clusters mean intensity, and r means pixel’s distance from a cluster’s centroid. The intensity of the ith pixel within a cluster is ${Z}_{i}$, ${C}_{k}$ is the center of the kth cluster, and ${x}_{i}$ is the intensity of the ith pixel. The number of pixels in a cluster is denoted by N. The COVID-19 segmentation process is depicted in Algorithm 1. Figure 4 illustrates the grouping of COVID-19 image data step by step.

**Figure:**
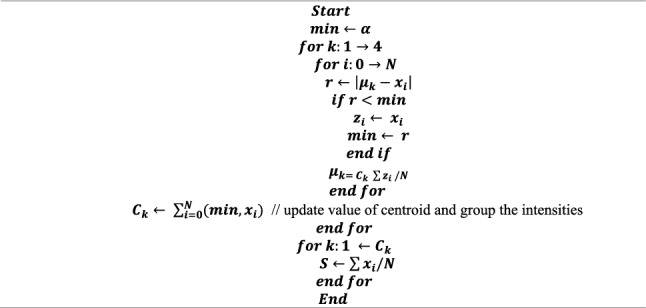
Algorithm 1: Proposed segmentation algorithm.

### Extraction of contourlet transform features

**Figure 5:**
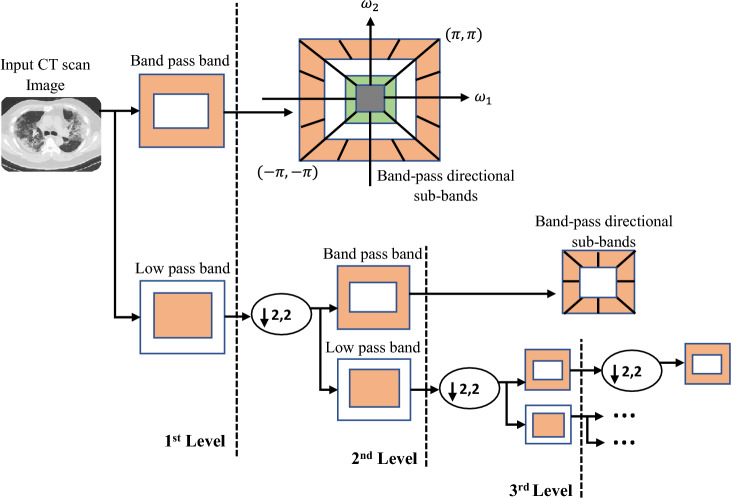
Overall structure of contourlet transform feature extraction method.

The contourlet transform tries to capture curves rather than points and includes anisotropy and directionality. The CT was created to solve the wavelet transform’s limitations such as poor directionality, shift sensitivity and lack of phase information^27^. At each scale, it allows for a variable and elastic number of directions while obtaining virtually critical sampling. The contourlet transform^28^ is accomplished based on two steps including Laplacian pyramid decomposition and directional filter banks (DFB). At every level of the Laplacian pyramid, a down-sampled lowpass version of the source image is generated, as well as the difference between the source image and the down-sample lowpass image, resulting in a high-pass image. The next level Laplacian pyramid builds an iterative structure linking with the down-sampled lowpass version of the original signal. DFBs are used to create high-frequency sub-bands with a variety of directions. The contourlet transform acts on two-dimensional CT scan images. This work generated sixteen different multi-directional multiscale images using four-level CT with the ‘9-7’ filter and computed thirteen various image features, including entropy, homogeneity, energy, correlation, and others from the segmented images, by enumerating the gray level co-occurrence matrix (GLCM) of each image. Figure 5 presents the contourlet transformed images considering edges, lines, textures and contours in contrast to the wavelet transform.

### Extraction of CNN based features

**Figure 6:**
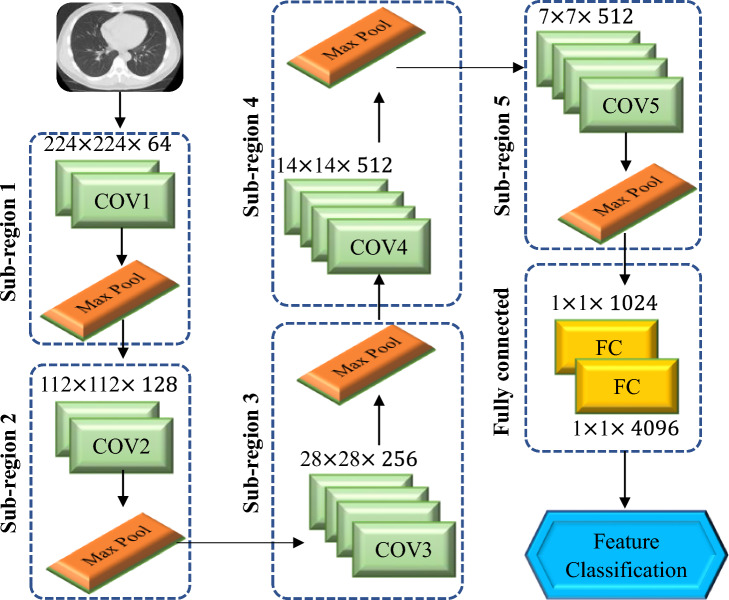
Architecture of VGG19 for feature extraction from CT scan images.

For feature extraction, the proposed system employed the benchmark VGG19 CNN model, which outperformed the other CNN models such as AlexNet, GoogleNet, and ResNet50. A 19-layer version of VGGNet^29^ was used to create this network. Figure 6 shows the VGG19 architecture, which includes sixteen convolution layers and three fully connected (dense) layers. For each convolution layer’s output, a non-linear ReLU was employed as an activation function. The entire convolution sections were divided into five sub-regions by five consecutive max-pooling layers. Two convolution layers were employed with depth dimensions of 64 and 128 respectively. Each of the other three sub-regions was made up of four consecutive convolution layers with depth sizes of 256, 512, and 512 in each sub-region. In this case, a convolutional kernel of size of 33 was chosen. The last layer of the proposed VGG19 models was replaced by a softmax classification layer. Two fully connected layers with neurons 1024 and 4096 were installed before the output layer. As a result, the fully connected layer yields 4096 features for classification.

### Features fusion and generation of optimized features

A fusion-feature vector was created by combining the extracted features from the contourlet transform and CNN. Overlapping, redundancy, and dimensional expansion are regular occurrences in all fusion-based techniques, therefore dimension reduction, as well as redundancy minimization or the elimination of irrelevant features, is required to obtain the optimum features. Many researchers obtain optimized features using Principal Component Analysis (PCA)^30^ and minimum Redundancy–Maximum Relevance (mRMR)^31^ but the BDE feature optimization method provides better performance than the others. For the dataset used in this study, three feature optimization approaches were tested and BED performed best.

In the mRMR feature selection algorithm, the mutual dependencies of x and y variable can be determined using Eq. (3) where p(x), p(y) and p(x,y) are the probability density functions.

3 $$I\left(x,y\right)= \iint p\left(x,y\right){\text{log}}\frac{p(x,y)}{p(x)p(y)}dxdy.$$

Equation (4) approximates the maximal relevance D(S,c), where x_i_ is the mean of all mutual dependencies and c is the class. As a result, the function R(S), is represented by Eq. (5) that can be used to add minimal redundancies. S is the feature combination.

4 $${\text{max}}D\left(S,c\right)= \frac{1}{\left|S\right|}\sum_{{x}_{i\in S, }}I\left({x}_{i, }c\right),$$

5 $${\text{max}}R\left(S\right)= \frac{1}{{|S|}^{2}}\sum_{{x}_{i}{{x}_{j}}_{\in S, }}I({x}_{i, }{x}_{j, }).$$

In the PCA algorithm, the covariance of features is determined to take uncorrelated features. PCA uses Eq. (6) to combine the correlated features.

6 $$\rho = \frac{\sum_{i=1}^{N}\left({X}_{i}-\overline{X }\right)({Y}_{i}-\overline{Y })}{n-1}.$$

The BDE feature selection technique is a heuristic evolutionary strategy for reducing the successive problem. The notion of advanced binary differential evolution (ABDE) is expanded to include feature selection difficulties. Three random vectors ${P}_{u1}$, ${P}_{u2}$, and ${P}_{u3}$ are chosen for vector pk for the mutation operation, so that u1 $\ne$ u2 $\ne$ u3 $\ne$ k, where k is a population vector arrangement. The dth characteristic of the difference vector (Eq. (7)) is zero if the dth dimensions of the vectors ${P}_{u1}$ and ${P}_{u2}$ are equal; otherwise, it has the same value as the vector ${P}_{u1}$:

7 $${difference\, vector}_{k}^{d}= \left\{\begin{array}{l}0, {P}_{u1}^{d}= {P}_{u2}^{d} \\ {P}_{u1, other}\end{array}\right\}.$$

Following that, the mutation and crossover processes are carried out, as illustrated by the Eqs. (8) and (9).

8 $${mute\, vector}_{k}^{d}= \left\{\begin{array}{l}1, {if\, different \,vector}_{k}^{d}= 1 \\ {{P}_{u3}^{d}}_{, other}\end{array}\right\},$$

9 $${W}_{k}^{d}= \left\{\begin{array}{l} {mute\, vector}_{k}^{d} , if y \le CR \left|d\right| d={d}_{random} \\ {{P}_{k}^{d}}_{, other}\end{array}\right\}.$$

Here, W denotes the try vector, ${CR}_{\epsilon }$(0, 1), a crossover amount, and ${\gamma }_{\varepsilon }$(0, 1) denotes the mutation amount. If the try vector ${W}_{k}$ has a higher fitness value than the current vector ${P}_{k}$, then it will be replaced in the selection phase. In a different way, the current vector ${P}_{k}$ is saved for the next generation. Finally, this fused method achieved 1300 accurate optimized features.

**Figure 7:**
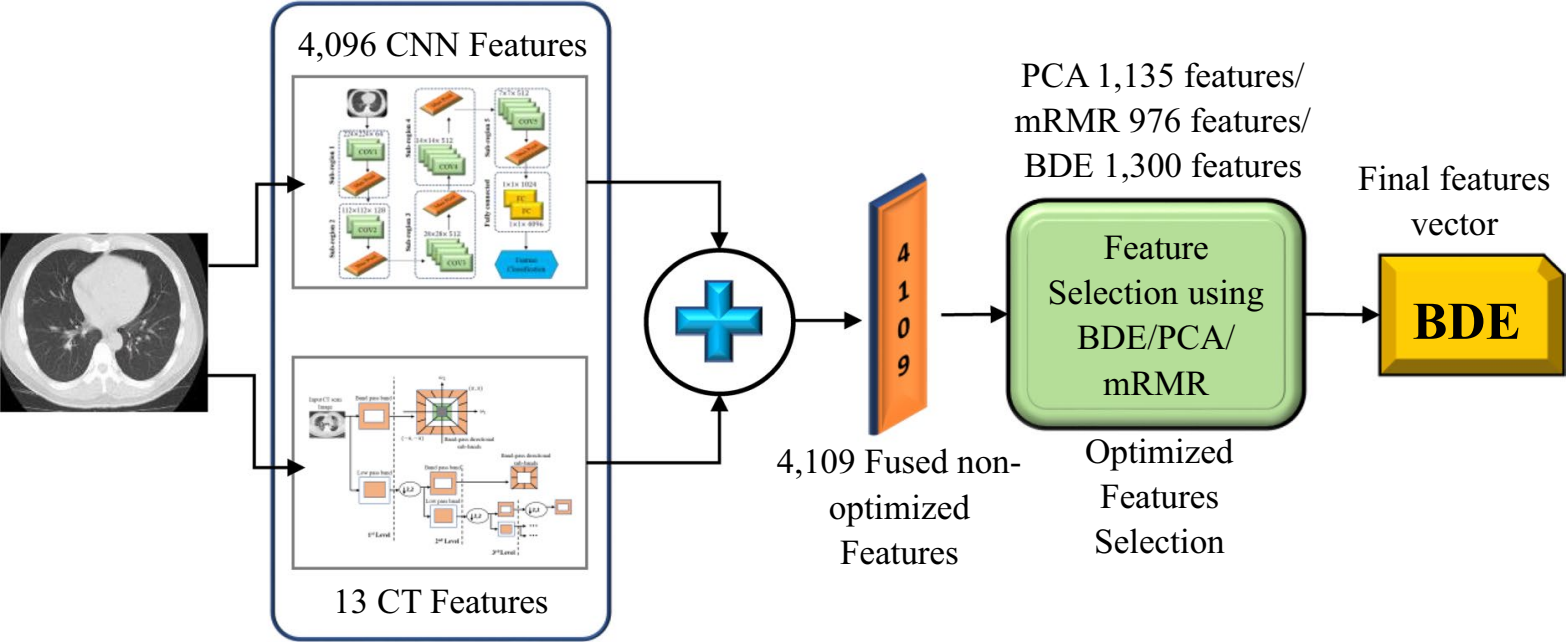
Block diagram of optimised feature selection process.

Figure 7 illustrates the steps in obtaining the optimized features in a single vector by fusing the features vectors extracted by the contourlet transform and CNN. The size of this feature vector is 4109. BDE based feature selection method was then employed to get 1300 most discriminating features.

### Hybrid selective mean filtering (HSMF) method

The authors suggested a novel, straightforward hybrid selective mean filter (HSMF) technique^32^ to calculate the average value selectively, unlike the traditional mean filter (MF) method, which calculates the average pixel utilizing all pixels in a given kernel region. A threshold value was used to define pixel selection (h). Noise was not considered in the noise reduction procedure if an adjacent pixel in a kernel was higher or smaller than the threshold value from the value of the core pixel. The pixel selection was performed with the following Eq. (10).

10 $${I}^{\prime}\left(x+i,y+j\right)= \left\{\begin{array}{l}I\left(x+i,y+j\right), \quad if \left|I\left(x,y\right)-I(x+i,y+j)\right|\le h\\ 0, \quad if \left|I\left(x,y\right)-I(x+i,y+j)\right|>h\end{array}\right..$$

If $\left|I\left(x,y\right)-I(x+i,y+j)\right|\le h, for every i and j$ then ${N}^{{{\prime}}}\left(x,y\right)=N-1.$ The noise image reduction is then calculated using Eq. (11).

11 $${I}_{SMF} \left(x,y\right)= \frac{{\sum }_{i=-\frac{n-1}{2},j=-\frac{m-1}{2}}^{+\frac{n-1}{2},+\frac{m-1}{2}}I^{\prime}(x+i,y+j)}{N^{\prime}(x,y)}.$$

**Figure 8:**
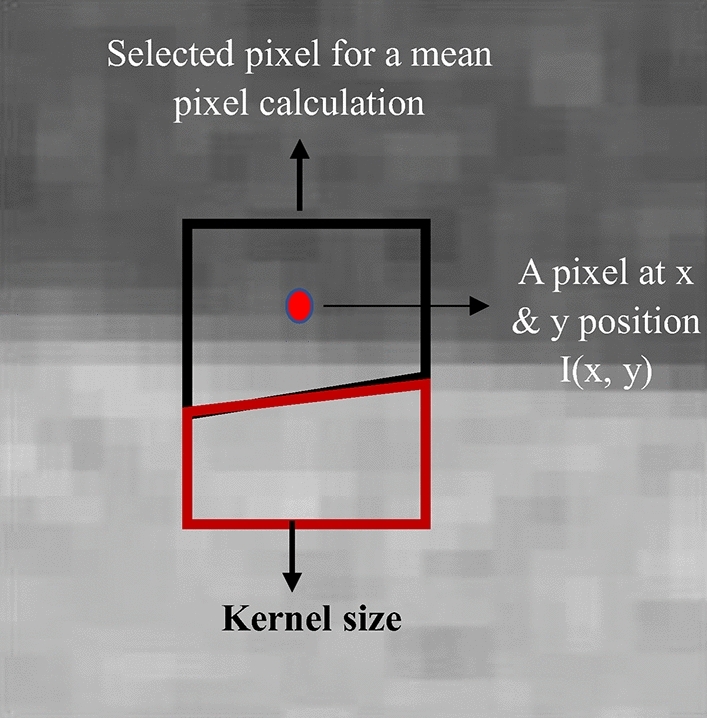
An illustration of picking neighboring pixels for noise reduction in the hybrid selective mean filter (HSMF) method.

In the Eqs. (10) and (11), the disparities between all nearby pixel values and the central pixel value are likely to exceed h in the edge areas. The pixel value ${I}_{SMF}$(x, y) is equal to I in this situation (x, y). In contrast, in the homogenous regions, the disparities between all nearby pixel values and the central pixel value are likely to be smaller than h. The pixel value ${I}_{SMF}$ (x, y) is equivalent to ${I}_{MF}$ in such situations (x, y). Figure 8 depicts the noise reduction process of the HSMF method. The mean pixel value at the central pixel in a position (x, y) was calculated only from the black area where the differences in pixel values from the value of the central pixel were less than the threshold value, not from all the pixels in a particular square kernel (i.e., union of black and red areas). The pixels outside of the black region, as well as those still inside the kernel of interest with pixel values higher than the threshold value, were not included in the calculation.

The threshold (h) was calculated using the magnitude of the standard deviation (SD) of the pixel values inside an image, which is a measure of noise^33^. To cover the majority of the image noise in this study, a 3 SD threshold was utilized. An approach proposed in Ref.^34^ was used to determine the SD automatically. This selects the minimum value of the standard deviation map automatically (SDM) as defined by Eq. (12).

12 $$SD=\mathrm{min}\left(SDM\right).$$

**Figure 9:**
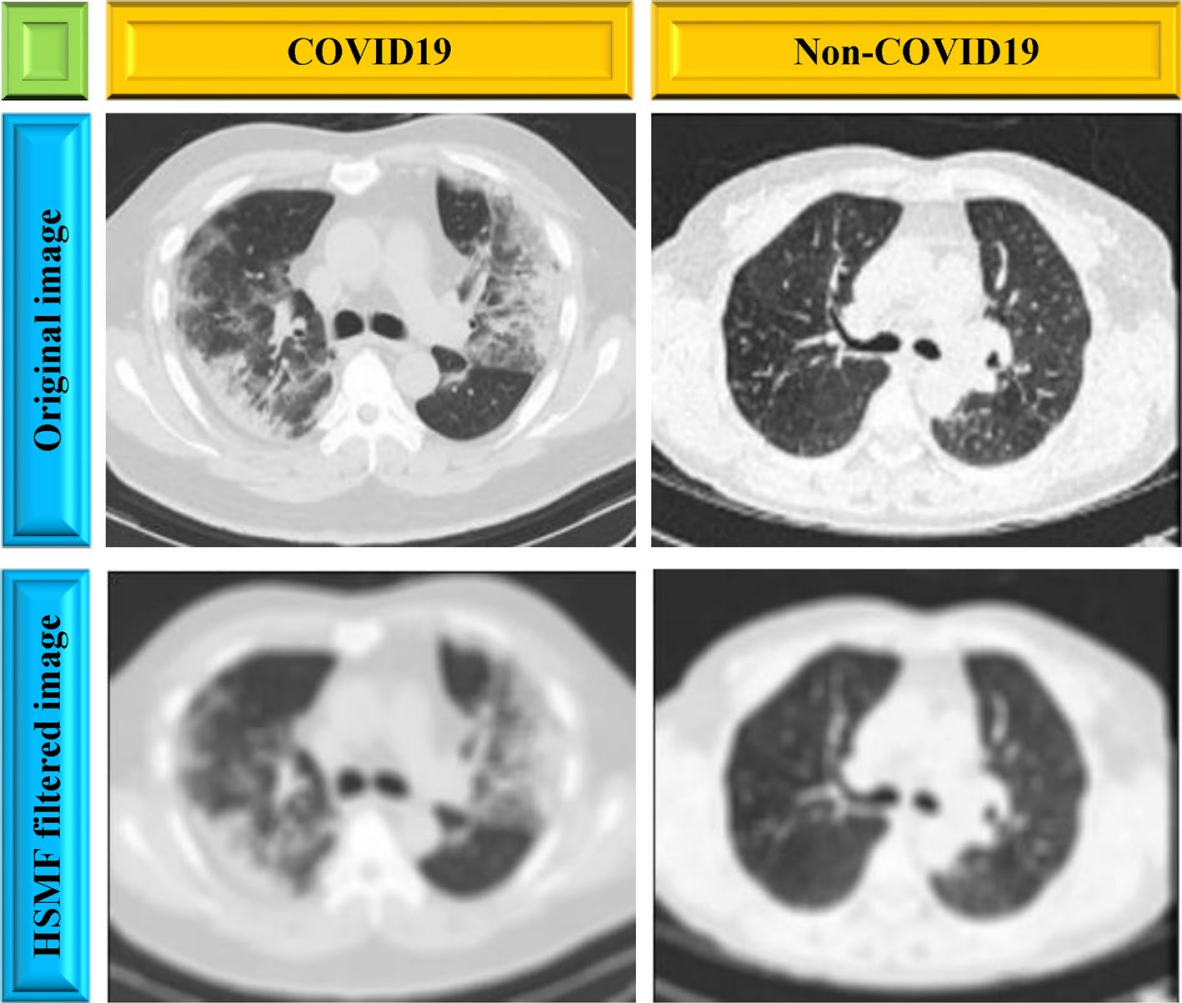
Filtered CT scan images using hybrid selective mean filter method.

The HSMF was supposed to reduce the noise dramatically while maintaining good spatial resolution. The technique is computationally light and fast as it is based on MF, making it easier to employ in clinical imaging than the BF (bilateral filter). Figure 9 displays the filtered image by using the HSMF method.

### Ensemble classifier

To determine the COVID-19, a ML/DL based ensemble classifier was employed^35^. Four ensemble models are commonly used to create the predictive classifier such as boosting, bagging, stacking, and voting^36^. The bagging approach of the ensemble methods like a bootstrap aggregation was used in this experiment. To compare the classification performance utilizing the optimized feature vector, three distinct types of classifiers including Long Short-Term Memory (LSTM), ResNet50 and Support Vector Machine (SVM) were employed. These three base classifiers were chosen as they typically outperform other ML/DL techniques. The categorization of any new instance by ensemble approaches is based on the classification votes of the basic classifiers. The output of each base classifier is regarded as a vote, with “v = 1” for the COVID-19 class and “v = 0” for the non-COVID-19 class.

The ensemble decision class is one that receives majority of the votes from the base classifiers that means $\left(if {\sum }_{i=1}^{n}v>\frac{n}{2}\right)$ as indicated in Eq. (13).

13 $$Ensemble\, Class= \sum\limits _{i=1}^{n}v,$$

where the total number of base classifiers is n.

**Figure 10:**
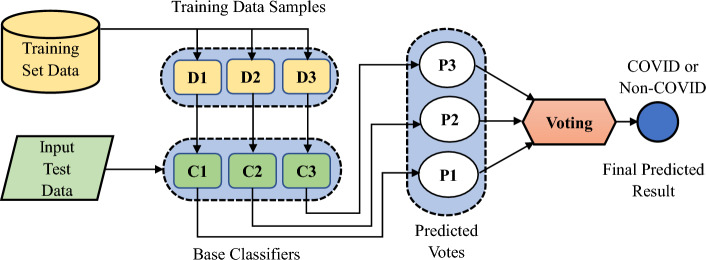
The bagging approach in the ensemble classifier.

Figure 10 represents the ensemble classifier-based bagging approaches where C1, C2, and C3 depict the LSTM, ResNet50, and SVM base classifiers, respectively. Similarly, P1, P2, and P3 signify the votes they represent. The final classification result combines the votes P1, P2, and P3 using Eq. (13) to yield the anticipated class based on the majority votes. To train the base classifiers, the training dataset set was divided into three subsets, D1, D2, and D3, then the testing was performed after training.

## Results and analysis of the proposed method

### Experimental settings

The training and testing procedures was set-up based on two classes: COVID-19 or non-COVID-19. The entire dataset was randomly partitioned with a ratio of 80:20 for the training and testing. All the training and testing were performed by MATLAB 2019b on a computer with an Intel Core i9, 3.0 GHz processor, and 8 GB RAM. The experimenting times were calculated using a GEFORCE RTX 2070 super GPU configuration. 4010 COVID-19 images and 7397 non-COVID-19 images are selected from the three standard datasets. The data distribution for developing COVID-19 detection framework is presented in Table 2.

**Table 2:** Data distribution for training and testing.

Datasets	COVID-19	Non-COVID-19
Training	3222	5935
Testing	788	1462
Total	4010	7397

### Evaluation metrics

Four metrics named accuracy (ACC), specificity (SP), sensitivity (SE), and precision (PR) were used to evaluate COVID-19 detection performance in the experiments as defined by Eqs. (14) to (17). The metrics were calculated from the confusion matrix. The proportion of patients correctly classified as COVID-19 and non-COVID-19 from all patients was measured by ACC. The capacity of a test to correctly identify patients with COVID-19 disease from correctly identified COVID-19 and incorrectly identified non-COVID-19 was measured by its sensitivity. The ability of a test to correctly identify patients without the COVID-19 condition from correctly identified non-COVID-19 and incorrectly identified COVID-19 patients was measured by its specificity. Precision refers to the percentage of correctly identified COVID-19 patients from all of the correctly and incorrectly identified COVID-19 patients.

14 $$Accuracy \, \left(ACC\right)= \frac{TP+TN}{TP+TN+FP+FN},$$

15 $$Specificity \, \left(SP\right)= \frac{TN}{TN+FP},$$

16 $$Sensitivity \, \left(SE\right)= \frac{TP}{TP+FN},$$

17 $$Precision \, \left(PR\right)= \frac{TP}{TP+FP},$$

where TP is true positive, TN is true negative, FP is false positive, and FN is false negative.

### Segmentation performance

In this work, evaluation metrics such as PSNR (peak signal to noise ratio), SSIM (structural similarity index), and RMSE (root mean square error) were calculated to measure the segmentation performance (Table 3). It was clear that the proposed modified region-based clustering method produced a better performance compared to the other segmentation methods in terms of PSNR and SSIM. However, the RMSE value was slightly worse than Fast C-means and K-means clustering methods.

**Table 3:** Comparison of several segmentation techniques.

Image segmentation techniques	PSNR	SSIM	RMSE
Threshold-based	27.89	0.8604	0.2890
Watershed method	31.83	0.8733	0.2578
C-means clustering method	33.75	0.8833	0.2645
Fast C-means clustering method	34.89	0.9089	0.2643
K-means method	35.11	0.9073	0.2641
Modified region-based clustering method (Proposed)	**36.17**	**0.9179**	**0.2645**

Best values are in bold.

### Filtered method performance

The HSMF has the potential to lower a given noise level by up to 75% without sacrificing spatial resolution. For a similar noise level, the bilateral filter (BF) was only able to lower the noise by 50% from 3.0 to 1.5 mGy. While at a higher noise level, BF cannot achieve a 50% reduction for instance a noise reduction from 6.0 to 3.0 mGy would not be possible. According to the current experiments, the HSMF reduced the noise by 75% (from 6.0 to 1.5 mGy), implying that the HSMF proved to be a better filter than the BF. In this study, PSNR was also used to compare the performance of different filtering techniques. Highest PSNR value was obtained for the HSMF (29.34) when compared to the adaptive median filter (AMF) (28.54) and the BF (28.75).

### Classification performance-feature fusion

The deep features from the pre-trained CNN (VGG19) model were additionally merged with the extracted features using the contourlet transform. While concatenating with deep CNN, the fusion of contourlet transform exhibited superior classification results than the interpolation-oriented descriptor such as scale-invariant feature transform (SIFT)^37^ and Histogram Oriented Gradients HOG^38^. Table 4 shows the comparison results utilizing contourlet transform, SIFT and HOG feature descriptors. It was clear that fusion of features by contourlet transform + CNN with feature optimization showed better performance that the individual techniques. Again, after optimization with three techniques (PCA, mRMR and BDE), BDE produce the best performance. Therefore, fusion of CNN features with contourlet transformed features and optimization with BDE were considered in this work. For all fused based CNN feature extractor models, this work employed the ensemble classifier.

**Table 4:** COVID-19 detection performance using features from several techniques.

Features	ACC	SP	SE	PR
SIFT only features	0.8556	0.8521	0.8492	0.8501
HOG only features	0.8617	0.8591	0.8512	0.8624
Contourlet transform only features	0.8763	0.8713	0.8892	0.8832
VGG19 only features	0.9766	0.9567	0.9678	0.9478
(Contourlet transform + VGG19) features without optimization	0.9802	0.9756	0.9848	0.9758
(Contourlet transform + VGG19) features with mRMR optimization	0.9827	0.9784	0.9871	0.9786
(Contourlet transform + VGG19) features with PCA optimization	0.9878	0.9869	0.9889	0.9876
(Contourlet transform + VGG19) features with BDE optimization	**0.9998**	**0.9993**	**0.9987**	**0.9987**

Best values are in bold.

### Classification performance-feature extraction by pre-trained CNN models

**Figure 11:**
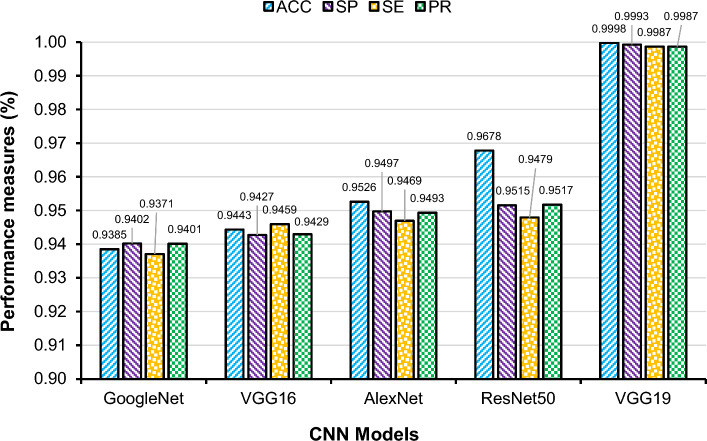
Comparisons of the classification performance results achieved by different CNN models for feature extraction and fusion combined with contourlet transform (feature selection by BDE optimisation and classification with ensemble technique).

Feature extraction experimentations were carried out by various pre-trained CNN models such as GoogleNet, VGG16, Resnet50, AlexNet and VGG19 and the extracted features were fused with features obtained by contourlet transform. It was identified that the VGG19 outperformed the others, in terms of all performance measures (Fig. 11). For each of the per-trained models, various performance metrics were determined, and the optimum results were obtained by changing the learning parameters and number of epochs. The best outcomes were achieved by selecting an appropriate learning parameter of 0.001 and an epoch of 50 for each of the pre-trained models.

### Classification performance-ensemble method

**Figure 12:**
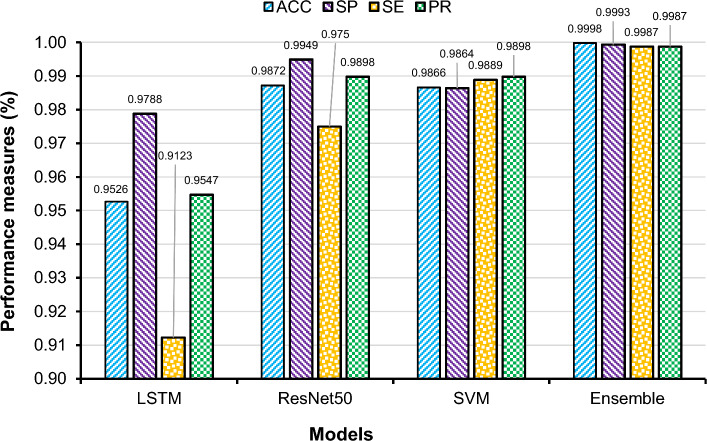
Comparison of the performances of individual and ensemble classifiers (feature extraction and fusion by VGG-19 and contourlet transform and feature selection by BDE optimisation).

**Figure 13:**
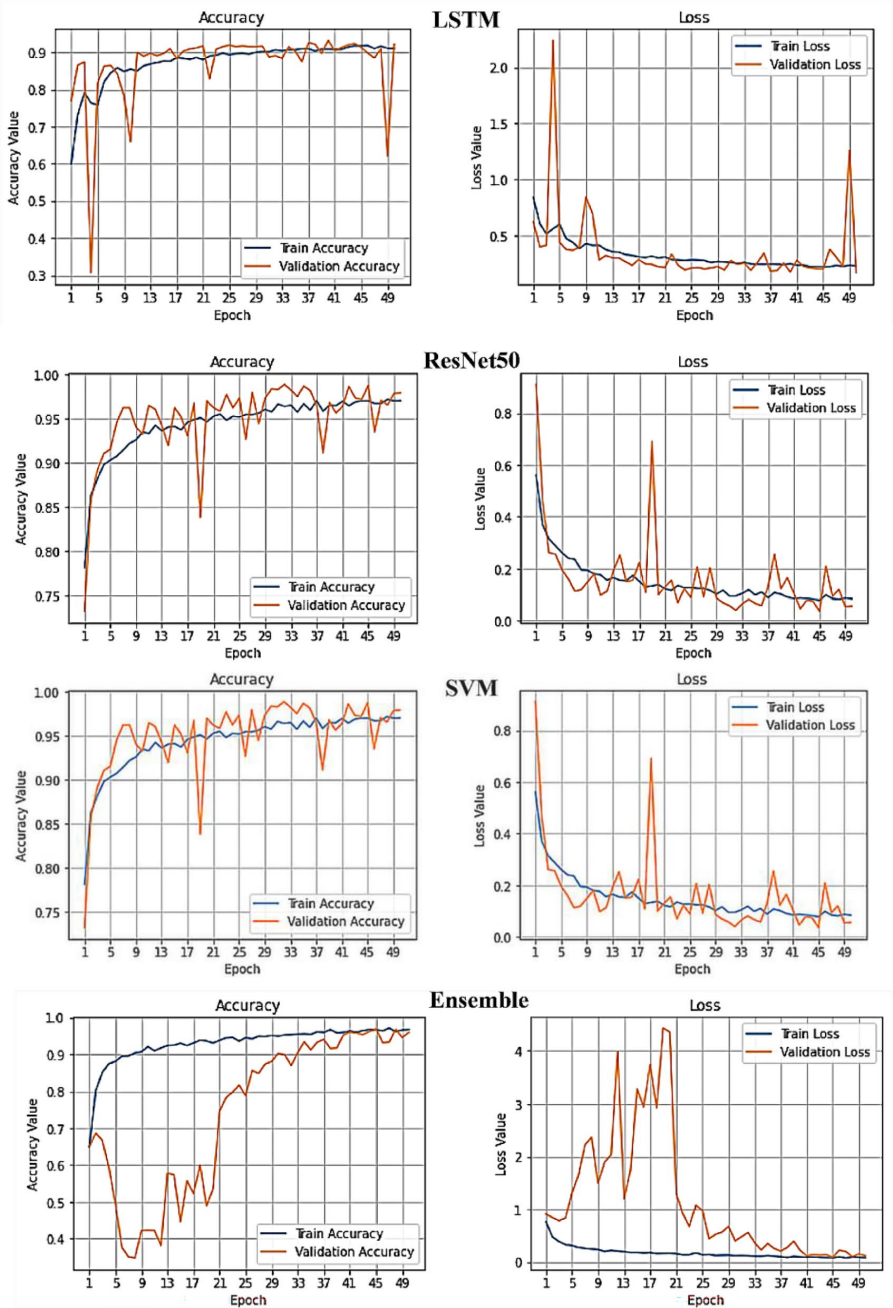
Comparison of training accuracy and loss performance curves for different classifiers.

For classification, the final feature vector with BDE optimization was considered as the input in developing the suggested ensemble model, which included LSTM, SVM and ResNet50 classifiers. Figure 12 shows the classification results of each separate classifier and the ensemble method. Compared to the three classifiers separately, the ensemble of these classifiers provided better outcome with an accuracy of 99.98%, a specificity 99.93%, a sensitivity and precision of 99.87%. Figure 13 shows different performance accuracy/loss curves for classification.

**Figure 14:**
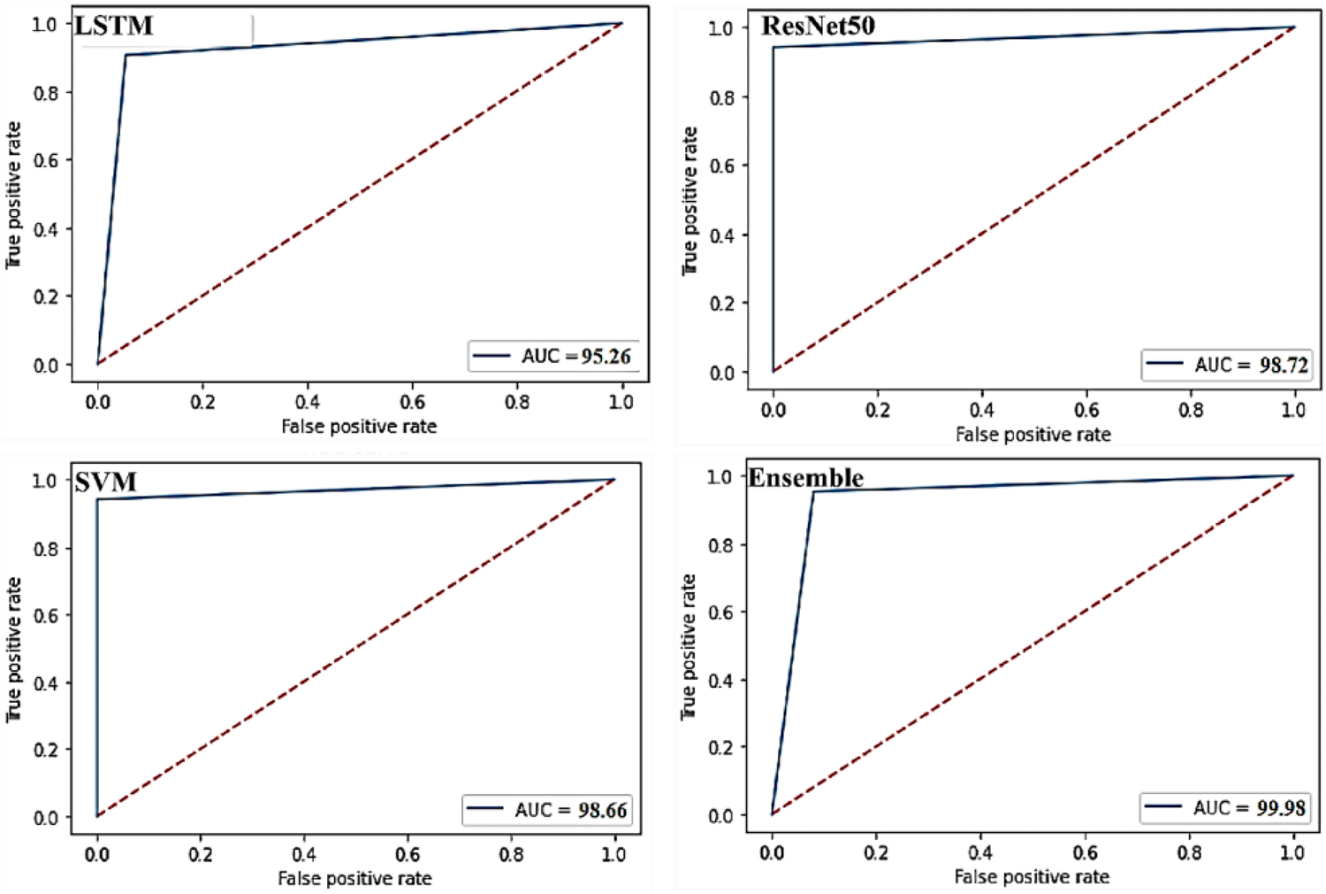
Receiver operating characteristics (ROC) curves for different models.

The true positive rate (TPR) against False Positive Rate (FPR) in a collection of threshold values is represented using a ROC curve. The Receiver Operating Characteristics (ROC) curves for the individual and ensemble methods are presented in Fig. 14. The goodness of the ensemble method’s classification performance was clearly noticed. The ROC curve’s area covered was almost 100% indicating that the model showed outstanding performance in terms of COVID-19 identification from the CT images.

**Figure 15:**
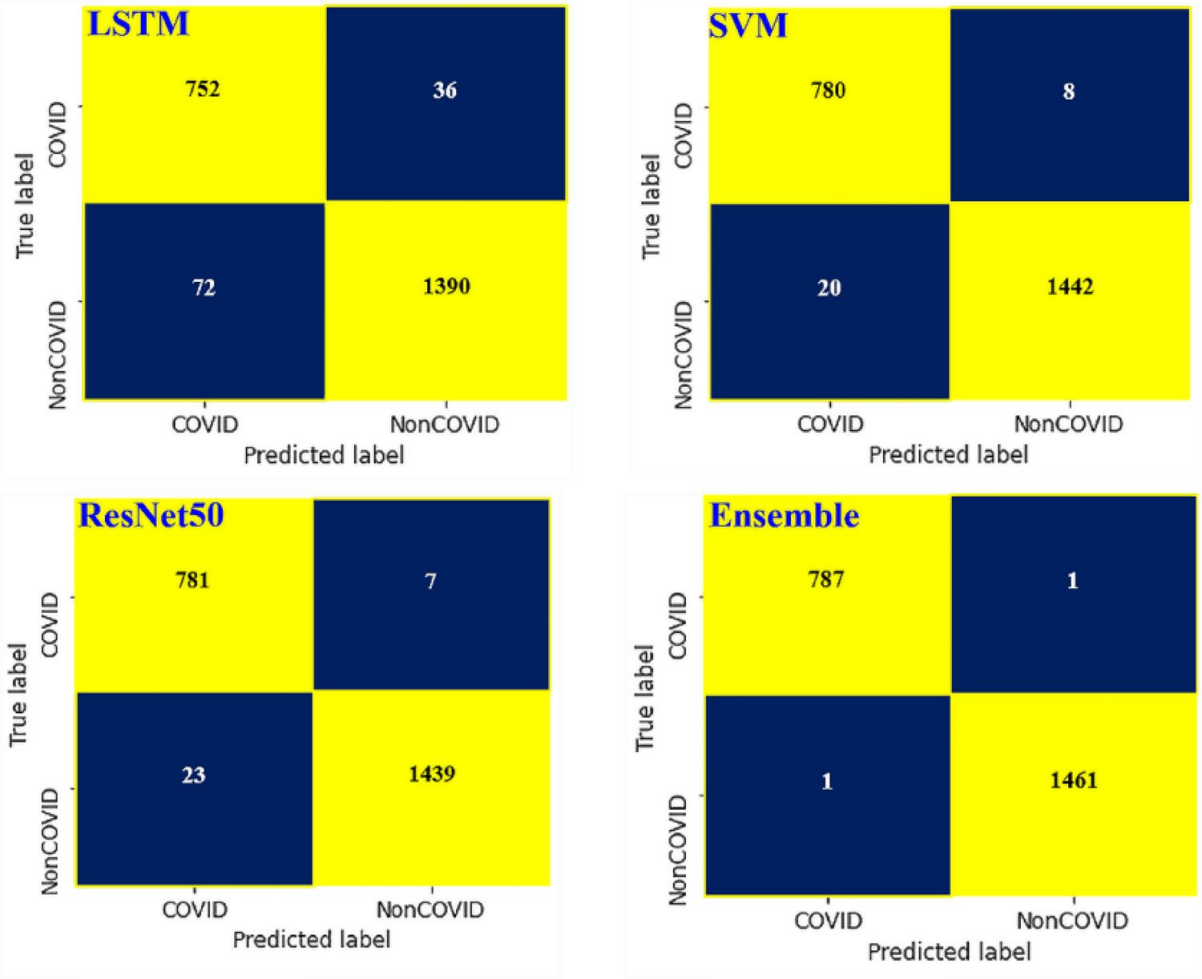
Confusion matrixes for different models.

Figure 15 presents two colors have been used in the confusion matrix based on the labels, represented by negative and positive prediction values. The yellow color represents (true prediction: TP and TN) how many COVID-19 and normal images have been detected accurately. Whereas the blue color (false prediction: FP and FN) indicates the number of COVID-19 and normal images that have been misclassified. According to the confusion matrix presented in, the proposed ensemble model missed 1 COVID-19 image (false negative) out of 788 COVID-19 images in this testing experiment, while it misidentified 1 non-COVID-19 images as COVID-19 images (false positive) out of 1462 non-COVID-19 images.

### Validation performance

The performance of the proposed model was additionally assessed using the generalization and k-fold validation techniques.

**Figure 16:**
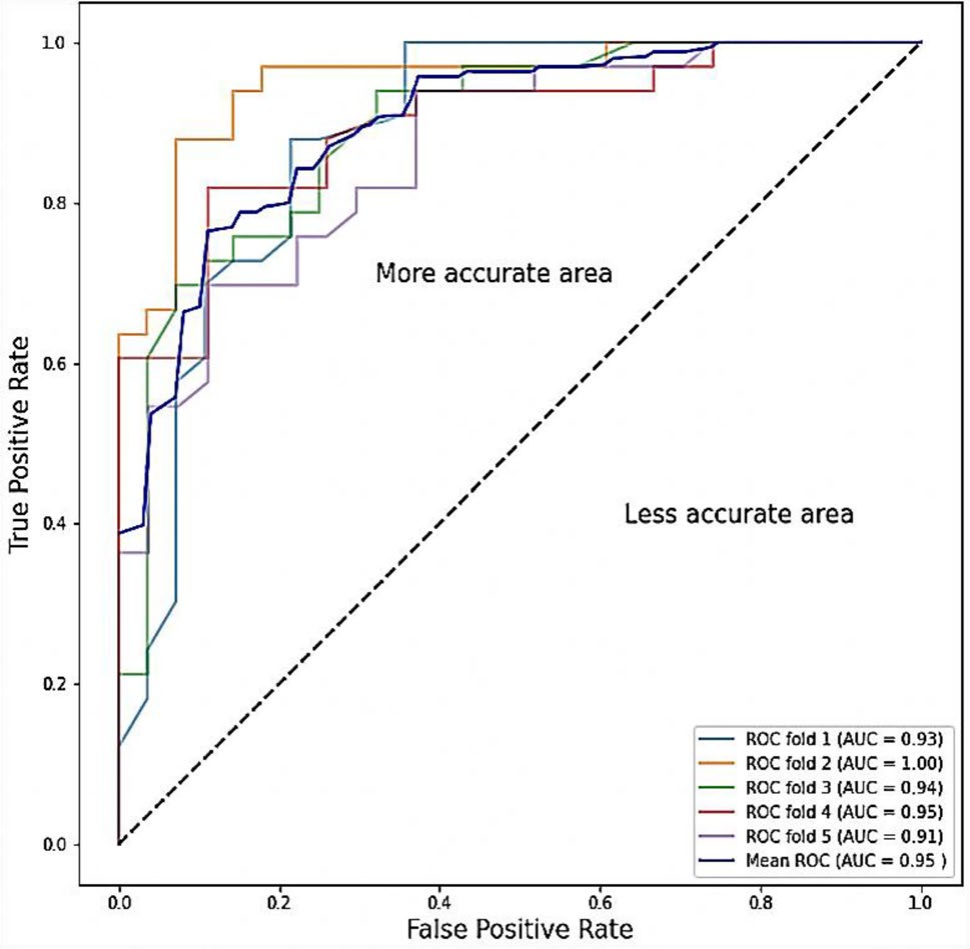
ROC curves for k-fold validation.

Cross-validation is a resampling method used in ML to ensure that a model is efficient and precise on unseen data. The K-fold cross-validation technique was employed in this study to divide the data into five folds and ensure that each fold was utilized as a testing set at least once. By doing so, the model was tested on completely unseen CT images, which would provide confidence in the model’s capacity to accurately recognize the COVID-19 cases. Table 5 presents the accuracy and loss values while testing and validating the proposed model using the fivefold cross-validation technique. It is clear that an average accuracy of 95% was obtained during testing and validation indicating the reliability of the proposed method on the unseen data. Figure 16 shows that ROC curves for individual and average folds.

**Table 5:** The values of accuracy and loss for training and test data during cross validation.

Fold	Accuracy		Loss	
	Training	testing	Training	testing
Fold1	0.9367	0.9236	0.0722	0.169
Fold2	0.9999	0.9789	0.0675	0.122
Fold3	0.9487	0.9685	0.0785	0.189
Fold4	0.9512	0.9479	0.0678	0.275
Fold5	0.9145	0.9574	0.0734	0.179
Mean	0.9568	0.9467	0.07188	0.934

**Figure 17:**
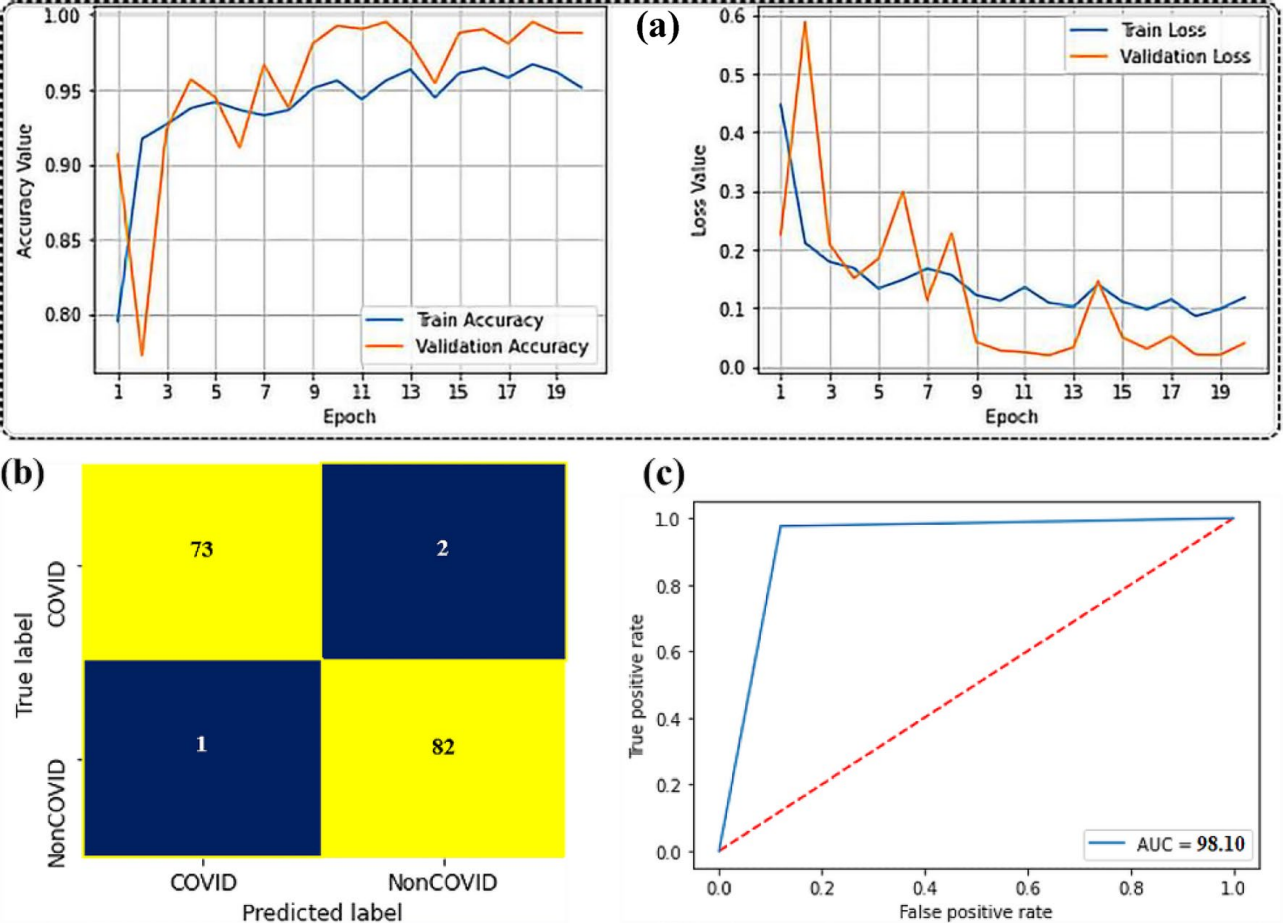
Training accuracy and training loss curves, confusion matrix, and ROC curves by appling generalization method. for COVID-19 Radiography database.

To determine the model’s performance, the generalization technique was used where the model trained on a given dataset would predict COVID-19 on a completely new dataset. The majority of the current research encounters challenges in using the generalization technique since the models were unable to recognize the varied relationship between pixel values in unique X-ray or CT images from different sources datasets^39^. In this study, COVID-19 Radiography database was considered for the generalization purpose which was entirely distinct from the training dataset^38^ and contained a total of 2541 images, of which 1200 were COVID19 and 1341 were non-COVID-19 cases. In this dataset, a total of 160 images were available for the testing purpose. The proposed system failed to identify 2 COVID-19 cases (false negative) out of 75 COVID-19 cases. Furthermore, out of 83 non-COVID-19 cases, it misidentified 1 non-COVID-19 cases (false positive) as the COVID-19 cases. Figure 17 illustrates the accuracy and loss performance by using generalization techniques along with confusion matrix and ROC curve for the COVID-19 Radiography database. The accuracy, precision, specificity, and sensitivity attained by the proposed model were 98.10%, 96.70%, 98.79%, and 97.33%, respectively, demonstrating the model’s robustness even when a new set of data was tested.

## Discussion

### Comparative analysis

Given the increasing size of biomedical datasets and complexity of the data, the use of ML and DL techniques in data analysis is continuing to grow in the coming years. As a result, novel strategies for uncovering the biological patterns, particularly biomedical imaging data, are required. This paper provides an ensemble classification technique for detecting COVID-19 cases from CT images. Table 6 compares the performance of the proposed strategy with the previous methods available in literature that used various classifiers and pre-processing techniques. It is obvious from the table that the proposed method outperformed all the previous state-of-the-art (SOTA) models by achieving a high classification accuracy of 99.98%.

**Table 6:** Performance comparison between the proposed method and existing methods.

Reference	Dataset size	Pre-Processing	Model	Accuracy (%)
Amine et al.^52^	1369	NA	Multi-task deep learning, U-Net segmentation	94.67
Ophir et al.^40^	947	NA	D and 3D Deep learning models, U-Net Mode	92.27
Wang et al.^53^	5340	Yes	Covid19-Net	81.24
Brunese et al.^41^	6523	Yes	Deep learning VGG16	97.00
Butt et al.^42^	306	Yes	Resnet18	98.20
Yang et al.^43^	295	NA	Dense Net	92.00
Jaiswal et al.^47^	2492	NA	DenseNet201	96.00
Ko et al.^48^	3993	Yes	Resnet50	99.54
Wu et al.^49^	495	Yes	VGG19	76.00
Mei et al.^50^	905	Yes	Inception_resnet_v2	95.00
Hasan et al.^45^	321	Yes	LSTM neural network classifier	99.68
Pathak et al.^51^	852	NA	Resnet50	93.01
Song et al.^44^	227	NA	BigBigGAN	92.00
Zain et al.^46^	1322	Yes	LSTM	98.00
Proposed method	11,407	Yes	Ensemble classifier	**99.98**

Best values are in bold.

Ensemble learning is a simple machine learning approach that seeks better predictive performance by combining the predictions from multiple models. However, in the proposed system, first, the dataset was pre-processed and segmented the Covid-19 affected regions using appropriate segmentation technique. Relevant features were extracted by two different feature extractors (VGG-19 and contourlet transform) and fused them in one vector. For classification purposes, the voting technique of the ensemble method was employed. It should be noted that the ensemble method was only used for classification of the features not fusing them together. Hence, in this proposed system, the modified region-based segmentation, fused features, BDE feature selection method, and ensemble classification play a significant role in obtaining significantly improved accuracy.

Most studies published in the literature did not use a segmentation technique to pre-process CT images^40–44^. However, the proposed method used modified region-based clustering technique for segmenting the COVID-19 CT images. Hasan et al.^45^ and Zain et al.^46^ used an LSTM network as a classifier to achieve a classification accuracy of above 98% on 321 and 1322 CT images, respectively. However, the LSTM networks might pose problems when training on small amounts of images since they are susceptible to overfitting. Also, the LSTM network requires additional memory and training time to train a network. Most previous research used a single pre-trained DL model as a classifier^41–52^, whereas LSTM, ResNet50 and SVM were combined as an ensembled classifier to achieve better classification performance. Most research only used a single dataset for their experiments^40,42–51,53^, making their models unreliable in predicting COVID-19 from a different dataset. Some work produced lower accuracies even when they used small number of datasets^40,43,44,49^. In contrast, the current method employing three distinct datasets with large number of images to develop the model would enhance its reliability.

### Complexity analysis of the proposed method

The processing time of a system plays a significant role in determining the image retrieval process. For this purpose, the entire operation of this study was performed by MATLAB 2019b on a high performance computer specified in “Experimental settings” section. The estimated processing times in this study are shown in Table 7. The entire operational time for each image is a combination of processing, training, and testing times. The processing time consists of preprocessing, feature extraction and classification times where the process begins with reading the image and finishes with feature extraction. On the other hand, the training time is the amount of time required to train each classifier on the complete dataset. The testing time merely consists of the prediction and voting of each classifier. Therefore, based on the processing times, it can be concluded that the proposed method was not computationally complex.

**Table 7:** The entire processing time of the proposed method.

Performed operation	Processing time (s)	Other info
Processing time (preprocessing + two feature extraction)	5.563764	For each image
Training time for the three classifiers	1.673428	For selected features
Testing time	0.023456	For each image

### Limitations and future work

This feature fusion ensemble method of detecting COVID-19 was developed based on three publicly accessible datasets. Despite huge success of the proposed method in identifying COVID-19 cases correctly, some drawbacks need to be highlighted for further improvement. One of the key challenges faced by the researchers in the ML based automated detection of COVID-19 cases is the requirement for a substantial annotated image dataset collected by a qualified physician or radiologists in order to develop a robust model.

To the best of our knowledge, the majority of the contemporary ML tools for medical imaging have this same constraint. The researchers are currently making their datasets available to the public in an effort to address this problem. However, the difficulty of gathering accurate data is made even more difficult by the absence of accurate annotation of the data that has already been collected.

Adopting zero-shot, few-shot, and deep reinforcement learning (DRL) techniques could help to address this problem in the near future^54,55^. Zero-shot learning has the capacity to build a recognition model for the unseen test samples that have not been labelled for training. Therefore, the zero-shot learning can address the issue of lack of training data for the COVID-19 classes. Additionally, a deep model can learn information from a small number of labeled instances per class using few-shot learning technique. On the other hand, DRL can reduce the need for precise annotations and high-quality images.

Another limitation is that in this study CT images were exclusively used. However, in future, the same described strategy can be applied on X-ray images to detect COVID-19 cases. This would enable to assess the effectiveness of THE model on a variety of image datasets. Although the proposed method achieved an outstanding performance on three publicly available dataset, the work has not been validated in actual clinical study yet. Therefore, efforts are required to test the model in clinical condition and gather feedback from the doctors and radiologists for further improvement of the model. In addition, fine-tuning of the proposed strategy could be carried out to address the issue of the lengthy training time resulting from the hybrid feature fusion technique.

## Conclusion

The proposed research has developed a high-accuracy, low-complexity intelligent ML model for COVID-19 identification using CT scan images. For the detection of COVID-19, the system combined the strength of contourlet transform with the power of CNN for feature fusion optimized by BDE, as well as the bagging-based ensemble classifier. The analysis of the results was performed considering the evaluation metrics including accuracy, sensitivity, specificity, and precision obtained from the confusion metrics. The proposed methods attained superior results of 99.98% accuracy compared to other classifiers including LSTM, ResNet50, and SVM or the existing approaches reported in the literature. Furthermore, the proposed system tested using fivefold cross-validation and with an unknown dataset for generalization purpose produced accuracies of 95.68% and 98.10% respectively.

## Data Availability

The datasets used and/or analysed during the current study available from the corresponding author on reasonable request.

## References

[1] Wiersinga WJ, Rhodes A, Cheng AC, Peacock SJ, Prescott HC (2020). Pathophysiology, transmission, diagnosis, and treatment of coronavirus disease 2019 (COVID-19): A review. JAMA.

[2] Cucinotta D, Vanelli M (2020). WHO declares COVID-19 a pandemic. Acta Biomed..

[3] Li J, Zhao G, Tao Y, Zhai P, Chen H, He H, Cai T (2021). Multi-task contrastive learning for automatic CT and X-ray diagnosis of COVID-19. Pattern Recogn..

[4] Kwekha-Rashid AS, Abduljabbar HN, Alhayani B (2021). Coronavirus disease (COVID-19) cases analysis using machine-learning applications. Appl. Nanosci..

[5] Hu B, Guo H, Zhou P, Shi Z-L (2020). Characteristics of SARS-CoV-2 and COVID-19. Nat. Rev. Microbiol..

[6] Shahid O (2021). Machine learning research towards combating COVID-19: Virus detection, spread prevention, and medical assistance. J. Biomed. Inform..

[7] Ghaderzadeh M, Asadi F (2021). Deep learning in the detection and diagnosis of COVID-19 using radiology modalities: A systematic review. J. Healthc. Eng..

[8] Wang MY, Zhao R, Gao LJ, Gao XF, Wang DP, Cao JM (2020). SARS-CoV-2: Structure, biology, and structure-based therapeutics development. Front. Cell. Infect. Microbiol..

[9] Polsinelli M, Cinque L, Placidi G (2020). A light CNN for detecting COVID-19 from CT scans of the chest. Pattern Recogn. Lett..

[10] Basu A, Sheikh KH, Cuevas E, Sarkar R (2022). COVID-19 detection from CT scans using a two-stage framework. Expert Syst. Appl..

[11] Kandati DR, Gadekallu TR (2023). Federated learning approach for early detection of chest lesion caused by COVID-19 infection using particle swarm optimization. Electronics.

[12] Karthik R, Menaka R, Hariharan M, Won D (2022). CT-based severity assessment for COVID-19 using weakly supervised non-local CNN. Appl. Soft Comput..

[13] Aversano L, Bernardi ML, Cimitile M, Pecori R (2021). Deep neural networks ensemble to detect COVID-19 from CT scans. Pattern Recogn..

[14] Zhao C, Xu Y, He Z, Tang J, Zhang Y, Han J, Shi Y, Zhou W (2021). Lung segmentation and automatic detection of COVID-19 using radiomic features from chest CT images. Pattern Recogn..

[15] Amyar A, Modzelewski R, Li H, Ruan S (2020). Multi-task deep learning based CT imaging analysis for COVID-19 pneumonia: Classification and segmentation. Comput. Biol. Med..

[16] He K, Zhao W, Xie X, Ji W, Liu M, Tang Z, Shi Y, Shi F, Gao Y, Liu J, Zhang J (2021). Synergistic learning of lung lobe segmentation and hierarchical multi-instance classification for automated severity assessment of COVID-19 in CT images. Pattern Recogn..

[17] Li C, Yang Y, Liang H, Wu B (2021). Transfer learning for establishment of recognition of COVID-19 on CT imaging using small-sized training datasets. Knowl. Based Syst..

[18] Mishra NK, Singh P, Joshi SD (2021). Automated detection of COVID-19 from CT scan using convolutional neural network. Biocybern. Biomed. Eng..

[19] Angelov P, Soares EA (2020). SARS-CoV-2 CT-scan dataset: A large dataset of real patients CT scans for SARS-CoV-2 identification. MedRxiv.

[20] Zhao, J. *et al*. Covid-ct-dataset: A CT scan dataset about covid-19. Preprint at http://arXiv.org/2003.13865490 (2020).

[21] Morozov, S. *et al*. *MosMedData: Chest CT Scans with COVID-19 Related Findings, v. 1.0*. https://mosmed.ai/datasets/covid19_1110 (2020).

[22] Atiyah IA, Mohammadpour A, Taheri SM (2018). Means: A fast fuzzy clustering. Adv. Fuzzy Syst..

[23] Caldairou B, Passat N, Habas PA, Studholme C, Rousseau F (2011). A non-local fuzzy segmentation method: Application to brain MRI. Pattern Recogn..

[24] Shah V, Keniya R, Shridharani A, Punjabi M, Shah J, Mehendale N (2021). Diagnosis of COVID-19 using CT scan images and deep learning techniques. Emerg. Radiol..

[25] Lehr JL, Capek P (1985). Histogram equalization of CT images. Radiology.

[26] Bhagwat K, More D, Shinde S, Daga A, Tornekar R (2013). Comparative study of brain tumor detection using K-means, fuzzy C means and hierarchical clustering algorithms. Int. J. Sci. Eng. Res..

[27] Fernandes FCA, van Spaendonck RLC, Burrus CS (2003). A new framework for complex wavelet transforms. IEEE Trans. Signal Process..

[28] Yang GW (2013). New feature extraction method based on contourlet transform for banknote classification. Appl. Mech. Mater..

[29] Ahmed S, Hossain T, Hoque OB, Sarker S, Rahman S, Shah FM (2021). Automated COVID-19 detection from chest X-ray images: A high-resolution network (HRNet) approach. SN Comput. Sci..

[30] Nandi D, Ashour AS, Samanta S, Chakraborty S, Salem MA, Dey N (2015). Principal component analysis in medical image processing: A study. Int. J. Image Min..

[31] Peng H, Long F, Ding C (2005). Feature selection based on mutual information criteria of max-dependency, max-relevance, and min-redundancy. IEEE Trans. Pattern Anal. Mach. Intell..

[32] Anam C, Adi K, Sutanto H, Arifin Z, Budi WS, Fujibuchi T, Dougherty G (2020). Noise reduction in CT images using a selective mean filter. J. Biomed. Phys. Eng..

[33] Anam C, Budi WS, Adi K, Sutanto H, Haryanto F, Ali MH, Fujibuchi T, Dougherty G (2019). Assessment of patient dose and noise level of clinical CT images: Automated measurements. J. Radiol. Prot..

[34] Anam C, Arif I, Haryanto F, Lestari FP, Widita R, Budi WS, Sutanto H, Adi K, Fujibuchi T, Dougherty G (2020). An improved method of automated noise measurement system in CT images. J. Biomed. Phys. Eng..

[35] Rokach L (2010). Ensemble-based classifiers. Artif. Intell. Rev..

[36] El-Melegy, M. T., Abo El-Magd, K. M., Ali, S. A., Hussain, K. F. & Mahdy, Y. B. Ensemble of multiple classifiers for automatic multimodal brain tumor segmentation. In *2019 International Conference on Innovative Trends in Computer Engineering (ITCE), Aswan, Egypt* 58–63. 10.1109/itce.2019.8646431 (2019).

[37] Wang, Z., Xiao, H., He, W., Wen, F. & Yuan, K. Real-time SIFT based object recognition system. In *2013 IEEE International Conference on Mechatronics and Automation* 1361–1366 (2013).

[38] Afshar P, Heidarian S, Enshaei N (2021). COVID-CT-MD, COVID-19 computed tomography scan dataset applicable in machine learning and deep learning. Sci. Data.

[39] Alam N-A, Ahsan M, Based MA, Haider J, Kowalski M (2021). COVID-19 detection from chest X-ray images using feature fusion and deep learning. Sensors.

[40] Ophir, G. *et al*. Rapid AI development cycle for the coronavirus (COVID-19) pandemic: Initial results for automated detection & patient monitoring using deep learning CT image analysis. In *Radiolo*

[41] Brunese L, Martinelli F, Mercaldo F, Santone A (2020). Machine learning for coronavirus covid-19 detection from chest X-rays. Procedia Comput. Sci..

[42] Butt C, Gill J, Chun D, Babu BA (2020). Deep learning system to screen coronavirus disease 2019 pneumonia. Appl. Intell..

[43] Yang S, Jiang L, Cao Z (2020). Deep learning for detecting corona virus disease 2019 (COVID-19) on high-resolution computed tomography: A pilot study. Ann. Transl. Med..

[44] Song Y, Zheng S, Li L, Zhang X, Zhang X, Huang Z, Chen J, Wang R, Zhao H, Chong Y, Shen J, Zha Y, Yang Y (2021). Deep learning enables accurate diagnosis of novel coronavirus (COVID-19) with CT images. IEEE/ACM Trans. Comput. Biol. Bioinform..

[45] Hasan AM, Al-Jawad MM, Jalab HA, Shaiba H, Ibrahim RW, Al-Shamasneh AR (2020). Classification of COVID-19 coronavirus, pneumonia and healthy lungs in CT scans using Q-deformed entropy and deep learning features. Entropy.

[46] Zain ZM, Alturki NM (2021). COVID-19 pandemic forecasting using CNN-LSTM: A hybrid approach. J. Control Sci. Eng..

[47] Jaiswal A, Gianchandani N, Singh D, Kumar V, Kaur M (2020). Classification of the COVID-19 infected patients using DenseNet201 based deep transfer learning. J. Biomol. Struct. Dyn..

[48] Ko H, Chung H, Kang WS (2020). COVID-19 pneumonia diagnosis using a simple 2D deep learning framework with a single chest CT image: Model development and validation. J. Med. Internet Res..

[49] Wu X, Hui H, Niu M (2020). Deep learning-based multi-view fusion model for screening 2019 novel coronavirus pneumonia: A multicentre study. Eur. J. Radiol..

[50] Mei X, Lee H-C, Diao K (2020). Artificial intelligence-enabled rapid diagnosis of patients with COVID-19. Nat. Med..

[51] Pathak Y, Shukla PK, Tiwari A, Stalin S, Singh S (2022). Deep transfer learning based classification model for COVID-19 disease. IRBM.

[52] Amine A (2020). Multi-task deep learning based CT imaging analysis for COVID-19 pneumonia: Classification and segmentation. Comput. Biol. Med..

[53] Wang S, Zha Y, Li W, Wu Q, Li X, Niu M, Wang M, Qiu X, Li H, Yu H (2020). A fully automatic deep learning system for covid-19 diagnostic and prognostic analysis. Eur. Respir. J..

[54] Karlos S, Mylonas N, Tsoumakas G (2021). Instance-based zero-shot learning for semi-automatic MeSH indexing. Pattern Recogn. Lett..

[55] Hua J, Zeng L, Li G, Ju Z (2021). Learning for a robot: Deep reinforcement learning, imitation learning, transfer learning. Sensors..

